# No association between TGF-β1 polymorphisms and risk of nasopharyngeal carcinoma in a large North African case-control study

**DOI:** 10.1186/s12881-016-0337-8

**Published:** 2016-10-12

**Authors:** Wafa Khaali, Khalid Moumad, El Khalil Ben Driss, Abdellatif Benider, Wided Ben Ayoub, Mokhtar Hamdi-Cherif, Kada Boualga, Elham Hassen, Marilys Corbex, Meriem Khyatti

**Affiliations:** 1 Oncovirology Laboratory, Institut Pasteur du Maroc, 1 Place Louis Pasteur, 20360 Casablanca, Morocco; 2 Departement of Biology, Faculty of Sciences, Abdelmalek Essaadi University, 93030 Tetouan, Morocco; 3 Service de Radiothérapie, Centre d’oncologie Ibn Rochd, 20360 Casablanca, Morocco; 4 Association Tunisienne de Lutte Contre le Cancer, 10006 Tunis, Tunisia; 5 Service d’épidémiologie, CHU de Sétif, 1900 Sétif, Algeria; 6 Service de Radiothérapie Oncologique, Centre Anti-Cancer de Blida, 09000 Blida, Algeria; 7 Molecular Immuno-Oncology Laboratory, Faculty of Medicine, Monastir University, 5019 Monastir, Tunisia; 8 Who Regional Office for Europe, Marmorvej 51, DK-2100 Copenhagen, Denmark

**Keywords:** Nasopharyngeal carcinoma, TGF-β1, Single nucleotide polymorphisms, Genetic susceptibility, North African population

## Abstract

**Background:**

Genetic susceptibility plays a key role in the development of nasopharyngeal carcinoma (NPC) and in fact the disease presents with an unusually high incidence in certain regions of the world like North Africa. We investigated the association between polymorphism of the Transforming growth factor-β1 (TGF-β1) and risk of NPC in North Africa. TGF-β1 is a multifunctional cytokine that acts as both a tumor suppressor and a stimulator of cancer development; it has been shown to influence risk of numerous other carcinomas including lung, breast and prostate cancer.

**Methods:**

TGF-β1 polymorphisms C-509T and T869C were studied in a large North African sample of 384 NPC cases and 361 controls, matched for age, sex and urban or rural residence in childhood. Genotypes were determined using polymerase chain reaction–restriction fragment length polymorphism.

**Results:**

No association was observed between individual single nucleotide polymorphisms or their haplotypes and NPC susceptibility (for TGF-β1 C-509T: OR = 0.74; 95 % CI 0.46 − 1.18; for TGF-β1 T869C: OR = 0.86; 95 % CI 0.56 − 1.31), even when the samples were stratified by age, gender and TNM stage.

**Conclusion:**

Contrary to what has been observed in Asian samples, in our North African sample, the TGF-β1 C-509T and T869C polymorphisms did not substantially influence NPC susceptibility.

**Electronic supplementary material:**

The online version of this article (doi:10.1186/s12881-016-0337-8) contains supplementary material, which is available to authorized users.

## Background

Nasopharyngeal carcinoma (NPC) is an epithelial malignancy arising from the mucosal epithelium of the nasopharynx [1]. It is a highly invasive and metastatic malignant tumor that differs significantly from other cancers of the head and neck in its distinct geographical distribution, causes, occurrence and clinical attributes [2, 3]. Both environmental factors (Epstein Barr virus & dietary factors) and genetic susceptibility play roles in the development of NPC [4, 5]. Although it is a rare malignancy in most parts of the world, NPC is endemic in a few well-defined populations such as those native in Southeast Asia, where the incidence is nearly 20–60 times higher than that reported in Western countries [6–8]. In North Africa, NPC is one of the most common head and neck cancers with an incidence rate around 3.5–10 per 100,000 inhabitants. In contrast to high-risk areas of Asia, the age-incidence curve in this intermediate-risk area is bimodal, with a first peak in the teens and a second at 45–60 years [9, 10].

Transforming growth factor-β1 (TGF-β1) is the most abundant isoform of the TGF-β and can regulate both immune system and cellular functions [11–13]. The TGF-β1 gene is located on chromosome 19q13.1–3 [14, 15]. Hundreds of single nucleotide polymorphisms have been reported for TGF-β1 and several of them are functional [16, 17]. The genetic variants of the TGF-β1 gene have been implicated in the susceptibility of a large range of carcinoma, including lung cancer [18], oropharyngeal cancer [19], prostate cancer [20] and breast cancer [21, 22]. The C-509T and T869C are the most commonly studied polymorphisms since it has been shown that they affect the expression of the TGF-β1 protein and gene–dose effects have been observed [23–25]. It has been reported that NPC patients display a lower level of TGF-β1 in plasma and a higher level in tumor tissues and surrounding stroma compared to healthy controls [26–28]. To date, three studies have investigated the association between TGF-β1 and NPC. They all focused on the C-509T and T869C polymorphisms and were all conducted in Chinese populations. In 2007, Wei et al. studying a sample of 108 NPC cases and 120 controls reported a significant increase of risk for the -509T allele (OR = 1.64) as well as for the 869C allele (OR = 1.70) [29]. In 2012, Hu et al. studying a sample of 522 NPC cases and 712 controls reported a significant decrease of risk for the −509T allele (OR = 0.67) and no association with the T869C polymorphism. Furthermore they showed that the −509C allele drive an ~1.7-fold increase of TGF-β1 expression in NPC cell lines [30]. In 2014, Qu et al. studying a sample of 194 cases and 231 controls did not report any significant association with NPC [31]. In view of these results it seemed relevant to study further the association of the TGF-β1 C-509T and T869C polymorphisms with NPC risk. The present study is the very first to investigate this association in the North African population.

## Methods

### Study population

Details of the sample used here, 384 NPC cases and 361 controls from the IARC (International Agency for Research on Cancer) international study of NPC are described elsewhere [4, 32]. Briefly, all cases and controls were recruited in the three North African countries Morocco, Algeria and Tunisia where NPC incidence is the highest. Inclusion criteria stipulated that all four grandparents of each subject were of Moroccan, Algerian or Tunisian origin in order to avoid all risk of admixture bias. The control group consisted of unrelated subjects frequency matched with cases on recruitment center, sex, age and household in childhood (urban or rural). Detailed demographic and epidemiologic data as well as blood samples were obtained from each subject at time of recruitment using a questionnaire designed at the International Agency for Research on Cancer. All subjects gave informed consent for the study, and the International Agency for Research on Cancer ethical committee approved the study.

### DNA extraction and genotyping

The peripheral venous blood was collected from all enrolled NPC patients and controls. Genomic DNA was extracted from the peripheral venous blood using the proteinase K digestion and phenol–chloroform extraction or using the Gentra Puregene Blood Kit.

The TGF-β1 C-509T and T869C genotypes were determined by using a polymerase chain reaction–restriction fragment length polymorphism method. As published by Kang et al. [18], the PCR primers for the C-509T (rs1800469) and T869C (rs1800470) polymorphisms were 5′- GGGTCCCTCTGGGCCCAGTT-3′ (forward) and 5′-GGGGGCAACAGGACACCCGA-3′ (reverse); and 5′-ACCACACCAGCCCTGTTC- 3′ (forward) and 5′-GATGGCCTCGATGCGCTT-3′ (reverse), respectively. The PCRs were performed in a total volume of 25 μl containing ~ 50 ng genomic DNA, 0.2 mM dNTP, 2.0 mM MgCl2, 0.25 μM each primer, and 1 unit of Taq polymerase (Go Taq DNA Polymerase, Promega). The PCR cycle conditions consisted of an initial denaturation step at 95 °C for 5 min followed by 35 cycles of 35 s at 94 °C; 30 s at 60 °C for C-509T and 58 °C for T869C; 30 s at 72 °C; and a final elongation at 72 °C for 7 min. The PCR products were incubated overnight at 37 °C with the appropriate restriction enzymes. The restriction enzymes for the C-509T and T869C genotypes were AvaI and MspA1I, respectively. The digested PCR products were resolved on 2 and 6 % agarose gel respectively and stained with Ethidium Bromide for visualization under U.V light.

### Quality control

A random sample representing 20 % of the cases and controls was blindly tested twice by different investigators, and the results were concordant for all duplicate sets.

### Statistical analysis

Genotype and allele frequencies of TGF-β1 were compared between NPC cases and controls using the Chi-squared (χ2) test, the odds ratios (OR) and 95 % confidence intervals (CIs) were calculated to assess the relative risk. Hardy-Weinberg equilibrium was tested for with a goodness of fit χ2 test with one degree of freedom to compare the observed genotype frequencies among the subjects with the expected genotype frequencies. The linkage disequilibrium (LD) between the polymorphisms was quantified using Haploview 4.2 software. The SPSS 20.0 statistical software package (Chicago, IL) was used for statistical analysis. The haplotypes and their frequencies were estimated using the HAPSTAT 3.0 software. Statistical significance was assumed at the *P* < 0.05 level. Power calculation was performed using the University of British Columbia tool for power calculation (http://www.stat.ubc.ca/~rollin/stats/ssize/).

## Results

The demographics of the cases and controls are shown in Table 1.

**Table 1:** Characteristics of the study population

Characteristics	Cases n (%)	Controls n (%)	*P-*value^a^
Whole sample	384	361	
Gender			
Male	263 (68.49)	243 (67.31)	0.731
Female	121 (31.51)	118 (32.69)	
Age mean (years)	42.24	43.58	
Age			
≤30	101 (26.30)	83 (22.99)	0.295
>30	283 (73.70)	278 (77.01)	
Country			
Tunisia	190 (49.48)	172 (47.65)	0.852
Morocco	180 (46.88)	174 (48.20)	
Algeria	14 (3.65)	15 (4.16)	
TNM stage^b^			
I	0	-	
II	13 (4.55)	-	
III	65 (22.73)	-	
IV	208 (72.73)	-	

^a^ *P*-value of χ2 comparing cases and controls

^b^TNM was missing for 98 cases out of 384

The genotypes and alleles frequencies of the TGF-β1 C-509T and T869C polymorphisms among the controls and the cases are shown in Table 2. Haplotype analyses were performed and the possible four haplotype frequencies are shown in Table 3. Genotypic distributions were in Hardy-Weinberg equilibrium in cases and in controls (*P* > 0.05) and the two polymorphisms were in relatively strong linkage disequilibrium (LD), |D′| = 0.65. None of the polymorphisms studied nor their haplotypes appeared to be associated with NPC risk.

**Table 2:** Genotype and allele distributions of TGF-β1 polymorphisms in NPC cases and controls. Odd Ratios obtained by univariate and multivariate analyses

Polymorphism	Cases n (%)	Controls n (%)	OR (CI 95 %)^a^	*P-*value	OR (CI 95 %)^b^	*P-*value
TGF-β1 −509						
Genotype						
CC	127 (39.20)	120 (37.03)	1.00	-	1.00	-
CT	153 (47.22)	148 (45.68)	0.97 (0.69–1.36)	0.891	0.97 (0.69–1.37)	0.879
TT	44 (13.58)	56 (17.29)	0.74 (0.46–1.18)	0.211	0.73 (0.45–1.17)	0.194
Allele						
C	407 (62.47)	388 (59.87)	1.00	-	1.00	-
T	241 (37.53)	260 (40.12)	0.88 (0.70–1.10)	0.278	0.87 (0.70–1.10)	0.251
TGF–β1 869						
Genotype						
TT	132 (36.57)	119 (35.52)	1.00	-	1.00	-
TC	165 (45.70)	149 (44.48)	0.99 (0.71–1.39)	0.992	0.99 (0.71–1.38)	0.955
CC	64 (17.72)	67 (20.00)	0.86 (0.56–1.31)	0.488	0.80 (0.52–1.23)	0.312
Allele						
T	429 (59.91)	387 (57.76)	1.00	-	1.00	-
C	293 (40.09)	283 (42.23)	0.93 (0.75–1.15)	0.530	0.92 (0.75–1.14)	0.453

*n* number of subjects

^a^Unadjusted Odds ratios and confidence interval for case-control comparison

^b^Odds ratios and confidence interval adjusted on age, sex and country

**Table 3:** Haplotype distribution of TGF-β1 polymorphisms in NPC cases and controls

Haplotype TGF-β1 −509–TGF-β1 869	Cases 2n = 600 (%)	Controls 2n = 598 (%)	OR (CI 95 %)^a^	*P-*value
C– T	295 (49.17)	304 (50.84)	1.00	-
T– C	190 (31.67)	183 (30.60)	1.07 (0.82–1.38)	0.608
C– C	69 (11.50)	60 (10.03)	1.18 (0.80–1.73)	0.382
T– T	46 (7.66)	51 (8.53)	0.92 (0.60–1.43)	0.738

*n* number of subjects

^a^Unadjusted Odds ratios and confidence interval for case-control comparison

As cases from the first age incidence peak (≤30) are believed to have a strongest genetic susceptibility, we tested association between NPC risk and TGF-β1 C-509T and T869C polymorphisms in the subgroups ≤30 and >30 year of age. No significant association was observed (Table 4).

**Table 4:** Distribution of TGF-β1 polymorphisms genotypes according to age and sex in NPC cases and controls

	≤30				>30			
Genotype	Cases n (%)	Controls n (%)	OR (CI 95 %)^a^	*P-*value	Cases n (%)	Controls n (%)	OR (CI 95 %)^a^	*P-*value
TGF-β1 −509								
CC	26 (30.23)	30 (38.96)	1.00	-	101 (42.44)	90 (36.44)	1.00	-
CT	42 (48.84)	31 (40.26)	1.56 (0.77–3.15)	0.211	111 (46.64)	117 (47.37)	0.85 (0.58–1.24)	0.392
TT	18 (20.93)	16 (20.78)	1.30 (0.55–3.05)	0.549	26 (10.92)	40 (16.19)	0.58 (0.32–1.02)	0.060
TGF-β1 869								
TT	32 (34.04)	23 (29.49)	1.00	-	100 (37.45)	96 (37.35)	1.00	-
CT	44 (46.81)	42 (53.85)	0.75 (0.38–1.49)	0.415	121 (45.32)	107 (41.63)	1.09 (0.74–1.59)	0.674
CC	18 (19.15)	13 (16.67)	0.99 (0.41–2.43)	0.992	46 (17.23)	54 (21.01)	0.82 (0.50–1.32)	0.414
	Men				Women			
Genotype	Cases n (%)	Controls n (%)	OR (CI 95 %)^a^	*P-*value	Cases n (%)	Controls n (%)	OR (CI 95 %)^a^	*P-*value
TGF-β1 −509								
CC	88 (40. 00)	82 (37.27)	1.00	-	39 (37.50)	38 (36.54)	1.00	-
CT	100 (45.45)	95 (43.18)	0.98 (0.65–1.48)	0.927	53 (50.96)	53 (50.96)	0.97 (0.54–1.75)	0.931
TT	32 (14.55)	43 (19.55)	0.69 (0.40–1.20)	0.190	12 (11.54)	13 (12.50)	0.90 (0.36–2.22)	0.818
TGF-β1 869								
TT	90 (36.00)	80 (36.20)	1.00	-	42 (37.84)	39 (34.21)	1.00	-
CT	105 (42.00)	94 (42.53)	0.99 (0.66–1.50)	0.973	60 (54.05)	55 (48.25)	1.01 (0.57–1.80)	0.965
CC	55 (22.00)	47 (21.27)	1.04 (0.63–1.70)	0.875	9 (8.11)	20 (17.54)	0.42 (0.17–1.03)	0.057

*n* number of subjects

^a^Unadjusted Odds ratios and confidence interval for case-control comparison

As NPC is more frequent among men than women [33, 34], we also stratified the analysis by sex but no association between the two polymorphisms and NPC risk was observed (Table 4).

Assuming that TGF-β1 plays a dual role in tumor suppression and oncogenesis [13, 35], we examined the association between the two TGF-β1 polymorphisms with TNM stage. However, no significant differences were found (Additional file 1: Table S1).

## Discussion

To date, the effect of the TGF-β1 gene on NPC susceptibility has been explored in only three case-control studies, all of them conducted in Chinese samples, but the results were conflicting. Wei et al. reported that the −509T and 869C alleles were associated with a significant increase of risk while Hu et al. [30] found that the −509T allele was associated with a reduced risk (and no association was observed with T869C). Finally, Qu et al. [31] found no association while they had the statistical power to find associations of the size of the ones described by Wei et al. [29].

In our North African sample, we did not find any significant associations between C-509T and T869C and risk of NPC, while we had the power to find association of the size of the ones reported previously. Our sample conferred 80 % power to detect OR above 1.37 or below 0.72 for the −509T allele and above 1.35 for the 869C allele.

The frequencies of the TGF-β1 alleles studied in our North African sample were quite similar to the one reported in the Chinese samples. Frequencies of the −509T and 869C were 0.401 and 0.422, respectively among our North African controls compared to 0.508 and 0.496 in Wei et al. control group, 0.478 and 0.488 in Hu et al. control group and 0.480 and 0.471 in Qu et al. control group [29–31]. These frequencies are higher than those observed among European Caucasians (0.110 and 0.180 respectively in a Spanish sample [36]; 0.102 and 0.171 in a Czech sample [37]). We also measured the LD between the two polymorphisms studied and found that its extent in control subjects in China [29] (**|**D′| = 0.914) was stronger than that in our study (|D′| = 0.65) and in Caucasian subjects from the UK (**|**D′| = 0.737) [38]. In addition, the frequencies of the two major haplotypes −509C/869T and −509T/869C among our controls were 0.508 and 0.306, respectively, which was relatively similar to those observed in the Chinese samples (0.429 and 0.433, respectively) [29] and the UK sample (0.640 and 0.240, respectively) [39].

Our study has both strengths and limitations. Its rigorous case-control design (with matching of cases and controls and check on the ethnicity of the four grand parents of each subject) and the detailed epidemiological and clinical evaluation are its main strength. The sample size is quite large in regards of the incidence of NPC in North Africa; 4 years of recruitment in six centers across three countries were necessary to gather the sample studied here. However, the sample size remains limited and it is possible that in a larger sample, and with more SNP’s studied, one could detect a significant association between TGF-β1 polymorphism and NPC risk.

To date studies of TGF-β1 C-509T and T869C polymorphisms effects on risks of cancers such as hepatocellular carcinoma [40, 41], breast cancer [22, 42], gastric cancer [43, 44], and esophageal squamous cell carcinoma (ESCC) [45, 46] have also yielded somewhat conflicting results. For example medium size studies of ESCC (including about 250 cases) performed in Chinese samples resulted for one in the 869C allele to be associated with increased risk of ESCC while C-509T was not associated [45] and for the other study, in T869C not to be associated while the −509T allele was associated with reduced risk [46].

Recruitment biases and small sample sizes may explain the discrepancies observed. However, variations in genetic backgrounds and environmental factors among populations might also generate discrepancies. The function of the TGF-β1 protein is complex and the true relationship between TGF-β1 and NPC may be modulated by gene-gene or gene-environment interactions. Cancer is a multifactorial disease and variations across populations in environmental exposure and genetic susceptibility may generate unclear results in association studies. The results obtained so far highlight the need to perform studies on large-size samples and in various geographic areas.

## Conclusions

Our results suggest that the TGF-β1 C-509T and T869C polymorphisms are not associated to NPC susceptibility in the North African population, or if such an association exists it is too weak to be detected with a sample of 384 cases and ﻿361 controls.

## Additional file

Additional file 1: Table S1. Association of TGF-β1 gene polymorphisms with TNM stage. (DOCX 21 kb)
