# Supplementary material for: No association between TGF-β1 polymorphisms and risk of nasopharyngeal carcinoma in a large North African case-control study
Source: BMC Med Genet. 2016 Oct 12;17:72. doi: 10.1186/s12881-016-0337-8 (PMC5062876; doi:10.1186/s12881-016-0337-8)
Supplement: Additional file 1: Table S1. — Association of TGF-β1 gene polymorphisms with TNM stage. (DOCX 21 kb) [file 12881_2016_337_MOESM1_ESM.docx]

**No association between TGF-β1 polymorphisms and risk of Nasopharyngeal Carcinoma**

**in a large North African case-control study**

**- Supplementary table -**

Wafa Khaali^1,2*^, Khalid Moumad^1^, El Khalil Ben Driss^2^, Abdellatif Benider^3^, Wided Ben Ayoub^4^, Mokhtar Hamdi-Cherif^5^, Kada Boualga^6^, Elham Hassen^7^, Marilys Corbex^8^ and Meriem Khyatti^1*^

^1^Oncovirology Laboratory, Institut Pasteur du Maroc, 20360 Casablanca, Morocco

^2^Departement of Biology, Faculty of Sciences, Abdelmalek Essaadi University, 93030 Tetouan, Morocco

^3^Service de Radiothérapie, Centre d'oncologie Ibn Rochd, 20360 Casablanca, Morocco

^4^Association Tunisienne de Lutte Contre le Cancer, 10006 Tunis, Tunisia

^5^Service d'épidémiologie, CHU de Sétif, 1900 Sétif, Algeria

^6^Service de Radiothérapie Oncologique, Centre Anti-Cancer de Blida, 09000 Blida, Algeria

^7^Molecular Immuno-Oncology Laboratory, Faculty of Medicine, Monastir University, 5019 Monastir, Tunisia

^8^Who Regional Office for Europe, Marmorvej 51, DK-2100 Copenhagen, Denmark

***Corresponding authors:**

Institut Pasteur du Maroc, 1 Place Louis Pasteur, 20360 Casablanca, Morocco

Phone: +212 5 22 43 44 57 / Fax: +212 5 22 26 09 57

**Email adresses:**

WK*: [wafa.khaali@gmail.com](mailto:wafa.khaali@gmail.com)

KM: [khalidmoum@gmail.com](mailto:khalidmoum@gmail.com)

EBD: [ebendriss@yahoo.fr](mailto:ebendriss@yahoo.fr)

AB: [beniderabdel@yahoo.fr](mailto:beniderabdel@yahoo.fr)

WBA: [wided.benayoub@rns.tn](mailto:wided.benayoub@rns.tn)

MHC: [hamdicherifm@ennour.org](mailto:hamdicherifm@ennour.org)

KB: kboualga2002@yahoo.fr

EH: elham_tn@yahoo.fr

MC: corbex.marilys@gmail.com

MK*: [meriem.khyatti@pasteur.ma](mailto:meriem.khyatti@pasteur.ma)

**Additional file 1: Table S1. Association of TGF-β1 gene polymorphisms with TNM stage.**

| **Genotype** | **Non-metastatic cases**  **(TNM I, II or III)** | **Metastatic cases**  **(TNM stage IV)** | **OR (CI 95%)*** | ***P-*value** |
| --- | --- | --- | --- | --- |
| **TGF-β1 -509** |  |  |  |  |
| CC | 30 (45.45) | 64 (37.20) | 1.00 | - |
| CT | 27 (40.90) | 84 (48.83) | 1.46 (0.79 – 2.69) | 0.228 |
| TT | 9 (13.63) | 24 (13.95) | 1.25 (0.51 – 3.01) | 0.619 |
| **TGF-β1 869** |  |  |  |  |
| TT | 28 (40.57) | 65 (32.82) | 1.00 | - |
| TC | 32 (46.37) | 91 (45.95) | 1.23 (0.67 – 2.23) | 0.507 |
| CC | 9 (13.04) | 42 (21.21) | 2.01 (0.86 – 4.68) | 0.105 |

n= number of subjects.

* Odds ratios and confidence interval for case-case comparison.
